# Arid1a-dependent canonical BAF complex suppresses inflammatory programs to drive efficient Germinal Center B cell responses

**DOI:** 10.21203/rs.3.rs-3871185/v1

**Published:** 2024-01-18

**Authors:** Ajay Abraham, Daniela Samaniego-Castruita, Jillian Paladino, Isabella Han, Prathyaya Ramesh, Mi Thao Tran, Rebecca M Southern, Ashima Shukla, Vipul Shukla

**Affiliations:** 1 Department of Cell and Developmental Biology, Northwestern University, Chicago, Illinois, USA, 60611; 2 Robert H. Lurie Comprehensive Cancer Center, Northwestern University, Chicago, Illinois, USA, 60611; 3 Center for Human Immunobiology, Northwestern University, Chicago, Illinois, USA, 60611

**Keywords:** B cells, Germinal Center, Epigenetics, BAF complex, Inflammation

## Abstract

Differentiating B cells in germinal centers (GC) require tightly coordinated transcriptional and epigenetic transitions to generate efficient humoral immune responses. The mammalian Brg1/Brm-associated factor (BAF) complexes are major regulators of nucleosomal remodeling, crucial for cellular differentiation and development, and are commonly mutated in several cancers, including GC-derived B cell lymphomas. However, the specific roles of distinct BAF complexes in GC B cell biology and generation of functional humoral immune responses are not well understood. Here, we show that the A–T Rich Interaction Domain 1a (Arid1a) containing canonical BAF (cBAF) complex is required for maintenance of GCs and therefore high affinity antibody responses. While Arid1a-deficient B cells undergo activation to initiate GC responses, they fail to sustain the GC program resulting in premature GC collapse. We discovered that Arid1a-dependent cBAF activity establishes permissive chromatin landscapes during B cell activation and is concomitantly required to suppress inflammatory gene programs to maintain transcriptional fidelity in early GC B cells. Interestingly, the inflammatory signatures instigated by Arid1a deficiency in early GC B cells recruited neutrophils and inflammatory monocytes and eventually disrupted GC homeostasis. Dampening of inflammatory cues with anti-inflammatory glucocorticoid receptor signaling rescued GC B cell differentiation of Arid1a-deficient B cells, thus highlighting a critical role of inflammation in impeding GC responses. In sum, our work identifies essential functions of Arid1a-dependent BAF activity in promoting efficient GC responses. These findings further support an emerging paradigm in which unrestrained inflammation limits GC-derived humoral responses, as reported in the context of severe bacterial and viral infections.

## Introduction

The Brg1/Brm-associated factor (BAF) complexes (also known as the Switch/Sucrose Non-Fermentable or SWI/SNF complexes) are important mediators of nucleosomal remodeling in eukaryotic genomes and play crucial roles in cellular differentiation through establishment of specific gene expression programs^1–4^. The BAF complexes are multi-protein assemblages composed of ~15 subunits that display tissue specific expression patterns, giving rise to extensive structural and functional diversity in their activity^2,5,6^.

In mammals, three main forms of BAF complexes have been described, the canonical BAF (cBAF), the polybromo-associated BAF (PBAF), and the non-canonical BAF (ncBAF) complex, which harbor distinct as well as overlapping subunits^2,7–9^. The cBAF, PBAF and ncBAF complexes, all contain one of the two mutually exclusive ATPase (catalytic) subunits, SMARCA4 (BRG1) or SMARCA2 (BRM) which can slide, evict or replace nucleosomes to remodel local chromatin states^1,2^. On the other hand, the AT-Rich Interaction Domain 1 (ARID1A and ARID1B), PBRM1 (and ARID2) and BRD9 proteins, are complex defining subunits of cBAF, PBAF and ncBAF complexes, respectively^1,2^. Within the cBAF complex, ARID1A and ARID1B are mutually exclusive paralogs harboring the AT-Rich Interaction Domain with putative DNA binding activity, which aid in targeting lineage determining and/or stimulus-responsive transcription factors to specific regions of the genome^1,2^. Notably, many components of BAF complexes have been shown to be recurrently mutated in several solid cancers and hematological malignancies, including mature B cell-derived lymphomas^2,10–15^. Among BAF subunits, *ARID1A*, *ARID1B*, and *SMARCA4* are each mutated in ~10% of Diffuse Large B-Cell Lymphomas (DLBCL), which are thought to originate from malignant transformation of germinal center B cells^12^.

Germinal centers (GC) are specialized micro-anatomical sites in the secondary lymphoid tissues where B- and T-cell interactions guide antigen-dependent clonal selection, immunoglobulin diversification, and affinity maturation to generate high affinity humoral immune responses^16,17^. The GC-specific epigenetic and transcriptional states are established through induction and concerted actions of several transcription factors and how their activities are coordinated on a genomic level continues to be an active area of investigation. Multiple different transcription factors and epigenetic modifiers are turned on at specific stages of B cell differentiation to direct early activation, GC initiation, expansion, and differentiation of memory B cells and plasma cells^16–18^. Although transcription factors are vital in establishing distinct transcriptional states in GCs, chromatin remodeling ensures systematic switching between these discrete transcriptional programs to navigate B cell differentiation^19,20^. The BAF subunits Brg1 and Baf155, which are common to all BAF complexes, have been shown to be essential for establishing B cell identity early during bone marrow development^19,20^. However, the functions of distinct BAF complexes in peripheral B cell responses are not well understood.

Since B cell differentiation is accompanied by extensive chromatin remodeling, and recurrent mutations in cBAF complex subunits are associated with GC-derived lymphomas^21,22^, here, we sought to investigate the role of Arid1a-dependent cBAF complex in GC biology. We show that Arid1a is required for GC B cell differentiation and generation of efficient humoral immune responses. Arid1a-dependent cBAF activity is essential for establishing accessibility at thousands of genomic sites during B cell differentiation and concurrently suppresses inflammatory gene programs to maintain transcriptional fidelity for GC B cell homeostasis. Intriguingly, genetic loss of Arid1a triggers an inflammatory milieu to cause premature GC collapse and dampening of these inflammatory signals rescues Arid1a-deficient GCs. These findings corroborate emerging paradigms highlighting detrimental effects of inflammatory states on GC reaction, commonly to patients with severe bacterial and viral infections^23–25^.

## Results

### Arid1a is required for efficient germinal center B cell responses

**Figure 1:**
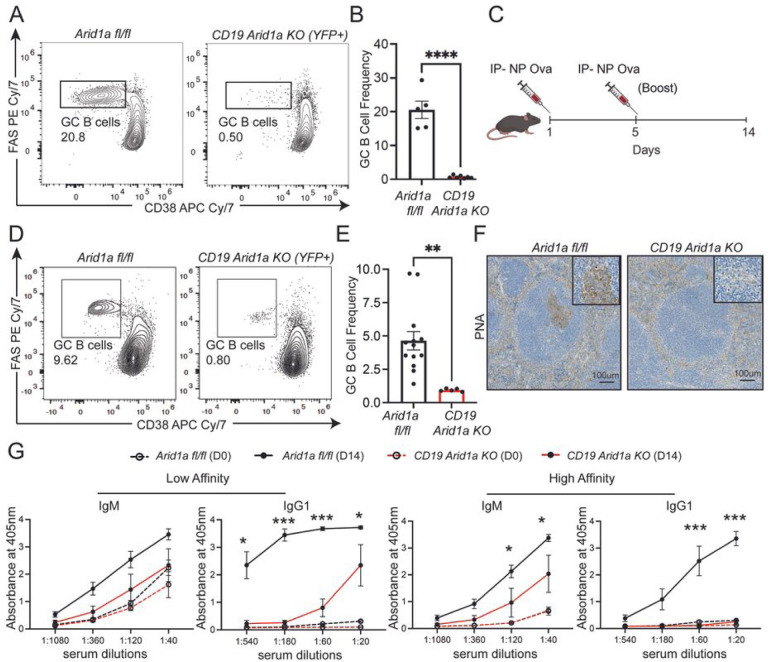
Arid1a deficiency in B cells perturbs germinal center B cell responses. **A** Representative flow cytometry analysis of GC B cells gated on B cells from Peyer’s Patches of *Arid1a fl/fl* and *CD19 Arid1a KO* mice. Numbers represent frequency of GC B cells, identified as FAS+ (y axis) and CD38low/- (x axis). **B.** Quantification of GC B cell frequency in Peyer’s Patches of *Arid1a fl/fl*(YFP-; n=5) and *CD19 Arid1a KO* (YFP+; n=7). **C.** Schematic for immunization with alum adjuvanted NP-Ova *for Arid1a fl/fl and CD19 Arid1a KO* mice. **D.** Representative flow cytometry analysis of splenic GC B cells gated on B cells from NP-Ova immunized *Arid1a fl/fl* and *CD19 Arid1a KO* mice at 14 days post-immunization. Numbers represent frequency of GC B cells. **E.** Quantification of GC B cell frequency in spleen of *Arid1a fl/fl* (YFP-; n=13) and *CD19 Arid1a KO* (YFP+; n=5). **F.**Representative immunohistochemistry images for PNA staining on formalin fixed spleen sections from *Arid1a fl/fl* and *CD19 Arid1a KO* mice immunized with NP-Ova at 14 days post immunization. **G.** Quantification of ELISA for detection of NP-specific low affinity (using NP-36) and high affinity (using NP-2) IgM and IgG 1 antibodies in the sera of NP-Ova immunized *Arid1a fl/fl* (n=3) and *CD19 Arid1a KO* (n=3). The serum antibodies were measured with 3-fold serial dilutions. Sera were collected prior to immunization Day 0 (DO) and at 14 days post-immunization (D14). Statistical significance is calculated using two-tailed Student’s t-test for B and E, multiple unpaired t-tests for G. Error bars represent mean ± standard error (s.e).; *p value < 0.05; **p value < 0.01; ***p value ≤ 0.0005; ****p value < 0.0001.

To study the role of Arid1a-associated cBAF activity in mature B cell development and function, we generated *CD19 cre Arid1a fl/fl* mice (*CD19 Arid1a KO*), wherein the *CD19 cre* transgene drives the expression of Cre-recombinase, robustly in mature B cells. We further crossed the *CD19 Arid1a KO* mice to carry a yellow fluorescent protein (YFP) reporter with *loxP-STOP-loxP* transcriptional stop cassette in the *Rosa26* locus (*Rosa LSL YFP*), which leads to YFP expression in a Cre-dependent manner. The Arid1a-deficient B cells (YFP+) from *CD19 Arid1a KO* mice showed no major alterations at early stages of development in the bone marrow (Figure S1A-B). In periphery, *CD19 Arid1a KO* mice showed efficient deletion of Arid1a protein in splenic B cells (Figure S1C), and this was associated with slight reductions in the frequency and absolute number of B cells (Figure S1D-F), but without any noticeable changes in the proliferation or survival of B cells at steady state (Figure S1G-J). Among distinct B cell subsets in the spleen, the distributions of immature and mature B cell subsets in *CD19 Arid1a KO* were comparable to those seen in *Arid1a fl/fl* control mice (Figure S2A-B). However, within the mature B cell compartment, the follicular B cell frequency was increased upon Arid1a deletion whereas the frequency of marginal zone B cells was reduced in comparison to Arid1a-proficient control B cells (Figure S2C-D). In addition to these changes, Arid1a deficiency was markedly associated with a near complete loss of Germinal Center (GC) B cells (Fas+CD38low) in the Peyer’s Patches (Figure 1A–B), which are lymphoid follicles in the small intestine where B cells undergo activation in response to intestinal microbiota and dietary antigens. The smaller proportions of GC B cells remaining in the Peyer’s Patches of *CD19 Arid1a KO* mice had very few cells expressing YFP, further implying a requirement for Arid1a-dependent cBAF complex activity in generation of GC B cells (Figure S2E).

Given the strong reduction of GC B cells in Peyer’s Patches, we next examined the role of Arid1a in induction of T cell-dependent GC B cell responses by immunizing the *CD19 Arid1a KO* and control (*Arid1a fl/fl* and *CD19* Cre) mice with hapten 4-hydroxy-3-nitrophenylacetyl (NP) conjugated to protein ovalbumin (NP-Ova) in alum as an adjuvant (Figure 1C). Similar to our observations in the Peyer’s Patches, at day 14 post-immunization with NP-Ova, the GC B cells (both frequencies and numbers) were clearly obliterated in *CD19 Arid1a KO* mice in comparison with control mice, as measured by flow cytometry and immunohistochemistry stain with peanut agglutinin (PNA, expressed on GC B cells) (Figure 1D–F). This was also accompanied by corresponding deficits in affinity maturation and generation of NP-specific high-affinity IgM and isotype class-switched IgG1 antibodies in sera (measured by ELISA) of *CD19 Arid1a KO* mice in comparison to controls (Figure 1G). Of note, the NP-specific IgM antibody production (both low and high affinity) was affected to a much lesser extent upon Arid1a deficiency, indicative of initial activation and differentiation at least in a subset of B cells from *CD19 Arid1a KO* mice.

**Figure 2:**
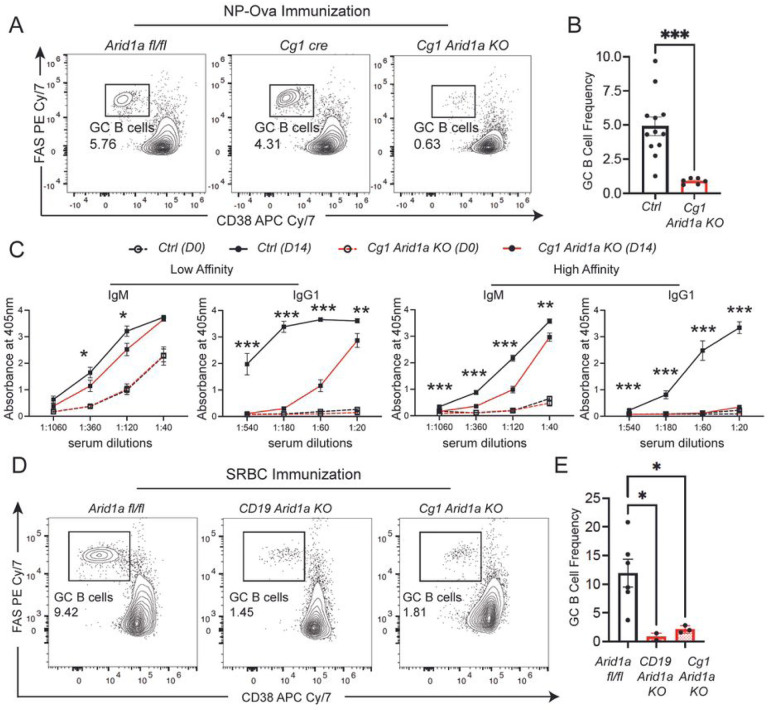
*Arid1a* is required for induction and maintenance of GCs. **A.** Representative flow cytometry analysis of splenic GC B cells from NP-Ova immunized *Arid1a fl/fl, Cg1 ere* and *Cg1 Arid1a KO* mice at 14 days post-immunization. **B.** Quantification of GC B cell frequency in spleen of *Control (Ctrl) (both Arid1a fl/fl and Cg1 cre)*and *Cg1 Arid1a KO* mice. **C.** Quantification of ELISA for detection of NP-specific low affinity (using NP-36) and high affinity (using NP-2) IgM and IgG1 antibodies in the sera of NP-Ova immunized *Ctrl* (*Arid1a fl/fl and Cg1 cre*; (n=6)) and *Cg1 Arid1a KO* (n=6). The serum antibodies were measured with 3-fold serial dilutions. Sera were collected prior to immunization Day 0 (D0) and at 14 days post-immunization (D14). **D.** Representative flow cytometry analysis of splenic GC B cells from SRBC immunized *Arid1a fl/fl, CD19 Arid1a KO* and *Cg1 Arid1a KO* mice at day 14 post-immunization. **E.** Quantification of GC B cell frequency in spleen of *Arid1a fl/fl, CD19 Arid1a KO* and *Cg1 Arid1a KO* mice. Statistical significance is calculated using two-tailed Student’s t-test for C, multiple unpaired t-tests for D and one-way ANOVAfor G. Error bars represent mean ± s.e.; *p value ≤ 0.05; **p value ≤ 0.01;***p value ≤ 0.0005; ****p value < 0.0001.

*CD19* driven Cre leads to Arid1a deletion in all mature B cells before their entry into the GCs. To further dissect the roles of Arid1a in initiation versus maintenance of the GCs, we crossed the *Arid1a fl/fl* mice to a mouse line harboring Cre-recombinase in the constant gamma 1 (*Cg1*) region of the immunoglobulin heavy chain locus (*Cg1 Arid1a KO*). The *Cg1 cre* drives Cre-recombinase expression following B cell activation and GC induction, which we confirmed using the *Rosa LSL YFP* reporter transgene (Figure S2F). NP-Ova immunization of *Cg1 Arid1a KO* mice, again led to a remarkable loss of GC B cells in comparison to *Cg1* Cre and *Arid1a fl/fl* control mice (Figure 2A–B). The *Cg1 Arid1a fl/*+ *(Cg1 Arid1a Het)* mice also showed a decrease in GC B cell differentiation to levels approximately half of that observed in control mice (Figure S2G). Furthermore, consistent with a lack of mature GCs, the affinity maturation, measured by serum levels of high affinity NP-specific IgG1 antibodies was once again severely impacted in *Cg1 Arid1a KO* mice compared with *Cg1 cre* and *Arid1a fl/fl* controls (Figure 2C).

GC responses to NP-Ova are pauci-clonal and restricted to a limited number of variable heavy and light chain clones, therefore, we used sheep red blood cells (SRBCs) to immunize *CD19 Arid1a KO, Cg1Arid1a KO*, and *Arid1a fl/fl* control mice with a more diverse, polyclonal antigen(s). The severe defects in GC B cell differentiation and IgG1 antibody production were still quite apparent even upon SRBC immunization in both *CD19 Arid1a KO and Cg1 Arid1a KO* mice compared with control mice (Figure 2D–E; S2H-J). Taken together, these studies clearly show that Arid1a is indispensable for the induction and maintenance of GC B cell responses.

### Arid1a-deficient B cells undergo activation and class switch recombination *in vitro*.

**Figure 3:**
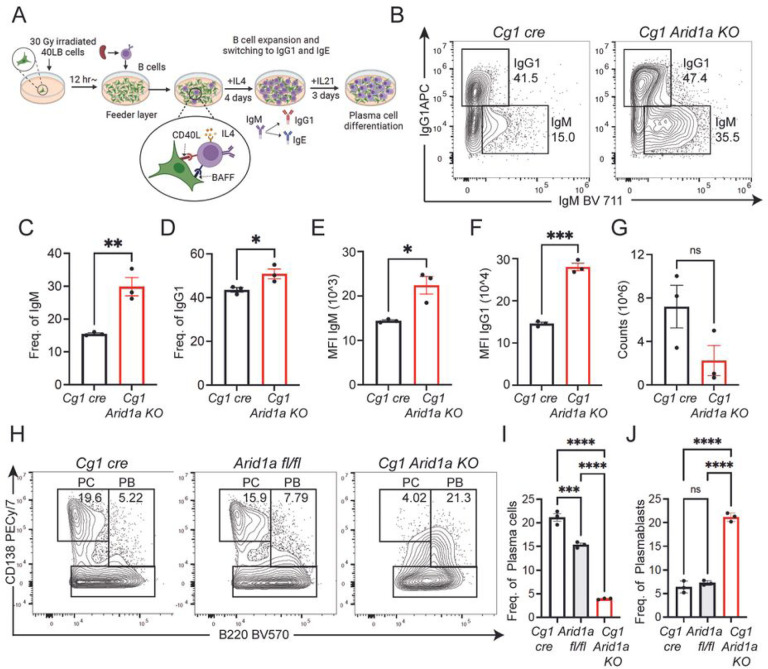
Arid1a-deficient B cells undergo activation *in vitro*. **A.** Schematic representation of the 40LB co-culture system for *in vitro* B cell stimulation. **B.** Representative flow cytometry analysis of IgG1 and IgM gated on B cells (*Cg1 cre and Cg1 Arid1aKO*) at day4 of 40LB co-cultures with IL4 (1ng/ml). **C and D.**Quantification of IgM and IgG1 frequencies at day 4 of 40LB co-culture. **E and F.** Median fluorescence intensity (MFI) of IgM and IgG1 ecll surface expression at day 4 of 40LB co-cultures. **G.** Cell counts of B cells from *Cg1cre* and *Cg1 Arid1aKO* co-cultured on 40LB cells for four days. **H.** Representative flow cytometry analysis of plasma cell (B220 low CD138 high) and plasmablast (B220+ CD138 intermediate) populations on 40LB co-cultures supplemented with IL21(10ng/ml) for 3 additional days after the initial 4-day co-culture in presence of IL4. **I.** Quantification of plasma cells and plasmablasts from *Arid1a fl/fl, Cg1 cre* and *Cg1 Arid1a KO* activated B cells (n=3). Statistical significance is calculated using two-tailed Student’s t-test for C-G, and one-way ANOVA for I and J. Error bars represent mean ± s.e.; *p value ≤ 0.05; **p value ≤ 0.01; ***p value ≤ 0.0005; ****p value < 0.0001.

We reasoned that the strong defects in Arid1a-deficient B cells to undergo GC B cell differentiation could simply be due to their inability to undergo activation. To test this, we adopted the CD40 ligand (CD40L) and B-cell activating factor (BAFF) expressing fibroblast feeder cells (40LB cells)^26^ to stimulate B cells in conditions mimicking *in vivo* GCs (Figure 3A). We isolated naive splenic B cells from Arid1a-deficient, *Cg1 Arid1a KO*, and *Cg1 cre* control mice and cultured them on irradiated 40LB cells, in presence of interleukin-4 (IL-4), which induced rapid activation, Cre expression, expansion and class switch recombination (Figure 3A–B, S3A-3B). To our surprise, Arid1a-deficient B cells underwent efficient activation (GL7+) and class switching to IgG1 isotype at levels similar, if not higher than those in control B cells (Figure 3B–D; S3A-B), 4 days post-stimulation on 40LB co-cultures. In addition, the cell surface expression of IgM and IgG1 isotypes in Arid1a-deficient B cells co-cultured with 40LB cells was much higher compared to control B cells, a feature also evident in Arid1a-deficient mature B cells *in vivo* (Figure 3E–F, S3C). While Arid1a-deficient B cells underwent efficient activation on 40LB feeder cells, the overall cell numbers of Arid1a-deficient B cells were noticeably (but not significantly) reduced in comparison with control B cells (Figure 3G). This decrease in cell numbers was not associated with obvious changes in cell proliferation (Ki67) or apoptosis (Cleaved Caspase 3) in Arid1a-deficient B cells (Figure S3D-G). Even Arid1a-deficient B cells from *CD19 Arid1a KO* mice, which lacked Arid1a prior to stimulation on 40LB cells, showed efficient activation and class-switch recombination to IgG1 in 40LB co-cultures, and displayed lower cell numbers but comparable cell proliferation and survival to control B cells (Figure S4A-H).

In comparison with control cells, our analysis revealed a reduction in differentiation to plasma cells in Arid1a-deficient B cells on 40LB co-cultures with IL-4 (Figure S3H-I, S4I-J). To examine this further, we cultured Arid1a-proficient and -deficient activated B cells on 40LB cells in the presence of Interleukin-21 (IL-21) which strongly promotes plasma cell differentiation. Arid1a-deficient B cells on 40LB cultures with IL-21, indeed exhibited a partial block in differentiation to plasma cells (B220 low CD138 high) at the plasmablast stage (B220+ CD138 intermediate) (Figure 3H–J). These results demonstrate that Arid1a-deficient B cells show reduced differentiation to plasma cells in 40LB co-cultures though are not inherently defective in their ability to undergo activation, expansion and class switch recombination.

### Arid1a deficiency is associated with induction of inflammatory molecular signatures in B cells

**Figure 4:**
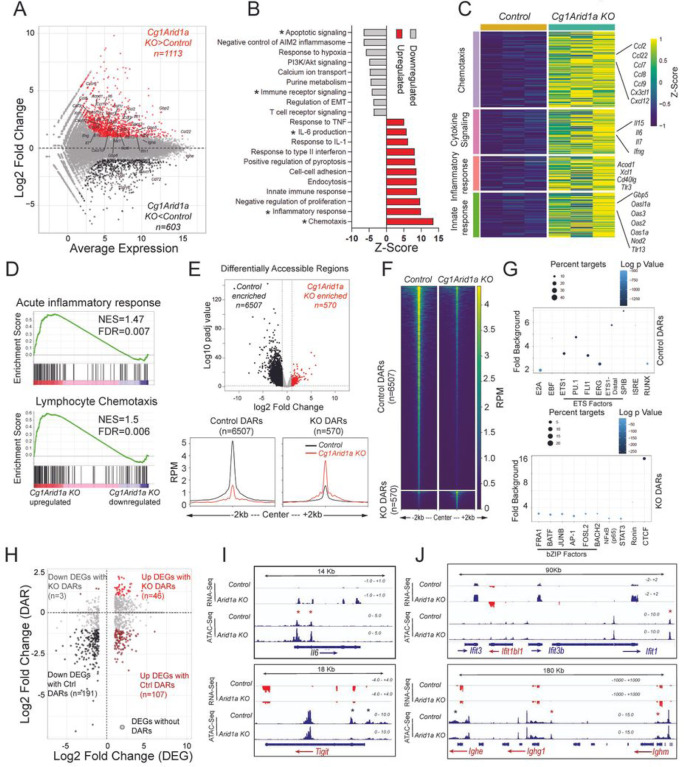
*Arid1a* deficiency is associated with induction of inflammatory signatures. **A.** MA plot of RNA-sequencing data displaying changes in gene expression in *Cg1 Arid1a KO* cells compared to control B cells. The highlighted genes (Red: upregulated, Black: downregulated) are differentially expressed genes. **B.** Upregulated (red) and downregulated (grey) pathways in *Cg1 Arid1a KO* compared to control B cells. The x-axis represents the *Z*-score, and asterisks highlight relevant pathways from Metascape analysis. **C.** Heatmap of gene expression data for *Cg1 Arid1a KO* and control B cells from chemotaxis, cytokine signaling, inflammatory response and innate response genes pathways. The color palette represents the Z-score from transcripts per million (TPM) values. **D.** Gene set enrichment analysis (GSEA) plots for the transcriptional profile of *Cg1 Arid1a KO* and control B cells, using gene sets from Acute inflammatory response (top) and Lymphocyte chemotaxis (bottom) pathways. Y-axis denotes enrichment score. NES, Normalized enrichment score, FDR, False discovery rate. **E.** *Top,* Volcano plot (top) of ATAC-seq data displaying differentially accessible regions (DARs) in *Cg1 Arid1a KO* compared to control B cells. *Bottom,* profile histograms showing the mean ATAC-seq signal in 6507 control and 570 KO enriched DARs (*control* B cell signal, black; *Cg1 Arid1aKO* Signal, red). **F.** Heatmap showing enrichment of ATAC-seq (average of 3 replicates) in *Cg1 Arid1a KO* and control B cells. Reads per million (RPM) values in DARs are plotted in +/− 2 kb windows from the center of the peak. **G.** Motif enrichment analysis of control DARs (top) and KO DARs (bottom). The *y-*axis indicates the fold enrichment over background, circle size indicates the percentage of regions with the respective motif, and the color indicates the significance (log_10_ *P* value). **H.** Scatter plot of log2 fold change DARs (*y-*axis) and gene expression changes (*x*-axis) between *Cg1 Arid1aKO* and *control* B cells. DEGs associated with a DAR are denoted in color (black: downregulated; red: upregulated in *Cg1 Arid1aKO*). **I.** Genome browser views of RNA-seq and ATAC-seq data showing gene expression and accessibility changes in *116* (top) and *Tigit* (bottom) loci. **J.** Genome browser view of RNA-seq and ATAC-seq data at *Ifit* (top) and *Igh* (bottom) loci. Asterisks denote changes in accessibility (red and black asterisks denote increased and decreased accessibility signals in *Cg1 Arid1a KO* cells, respectively). Please see bioinformatics analysis in methods for additional information.

To characterize the molecular features of B cells lacking Arid1a, we performed RNA sequencing (RNA-Seq) and Assay for Transposase-Accessible Chromatin using sequencing (ATAC-Seq) for genome-wide transcriptomic and chromatin accessibility measurement of control and Arid1a-deficient B cells. We stimulated control (*Cg1 cre)* and Arid1a-deficient (*Cg1Arid1a KO*) B cells on 40LB co-cultures with IL-4 for four days (in triplicates) and harvested activated B cells (following 40LB cell depletion). Interestingly, the transcriptomic profiling revealed approximately twice as many upregulated (n=1113) than downregulated (n=603) genes in Arid1a-deficient B cells compared with control cells (FDR ≤ 0.05; log2 fold change greater than +/− 1) (Figure 4A). This was somewhat surprising considering that ATAC-seq analysis highlighted a global decrease in chromatin accessibility upon Arid1a deletion in B cells. The downregulated genes in Arid1a-deficient B cells encompassed pathways involved in negative regulation of apoptotic and B cell receptor signaling (*CD72, Tigit, Spry2, Havcr1*) (Figure 4A), whereas the upregulated genes were mainly categorized into chemotaxis, innate immune response, pyroptosis and inflammatory response pathways linked with response to IL-6, interferons and tumor and necrosis factor (TNF) signaling (Figure 4A–B). Indeed, a number of chemokine (*Ccl2, Ccl3, Ccl6, Ccl7, Ccl8, Ccl9, Ccl12, Ccl22, Cxcl10, Cxcl12, Xcl1*), chemokine receptor (*Ccr1, Csf1r, Csf3r, Cxcr3, Cxcr6, Tnfrsf1a, Tnfrsf12a, Ltbr*), cytokine (*Il6, Ifng, Il15, Il7*) and interferon stimulated (*Mx1*, *Ifit1, Ifit3, Ifitm3, Ifih1, Gbp5, Gbp6, Gbp7*) genes were strongly upregulated in Arid1a-deficient B cells (Figure 4C). Gene set enrichment analysis (GSEA) of differentially expressed genes also revealed core enrichment of acute inflammatory response, lymphocyte chemotaxis, innate immune response, IL-6 production and pyroptotic gene signatures, emphasizing the enrichment of these pathways upon Arid1a deficiency (Figure 4D; S5A-C).

ATAC-Seq profiling revealed 7077 differentially accessible regions (DARs) (FDR ≤ 0.05) in Arid1a-deficient B cells compared to controls. The majority of DARs (n=6507) showed a reduction in chromatin accessibility upon Arid1a loss (referred to as Control enriched DARs), consistent with an essential role for Arid1a-dependent cBAF activity in opening regions of closed chromatin. However, a small number of DARs (n=570) also displayed an increase in accessibility in Arid1a KO B cells, which we refer to as KO enriched DARs (Figure 4E–F, S5D). The control DARs were more distally located to the transcription start sites (TSS), with a median distance of ~23kb. Whereas the KO DARs were located closer to the promoters and TSSs, with a median distance of ~13Kb and in fact, ~26% of KO enriched DARs were located within +/− 1Kb of the TSS. Moreover, the KO enriched DARs exhibited a distinctive accessibility pattern in Arid1a-deficient B cells, characterized by “a nucleosomal laddering effect” around the center of the peaks (Figure 4E). The control DARs mainly harbored DNA motifs recognized by E2A, EBF, ETS-family (PU.1, SPIB, ETS1, ERG, FLI1), ETS:IRF, IRF and Runx family of transcription factors, which are required for B cell activation and differentiation, suggesting important functions of Arid1a-dependent BAF activity in opening genomic regions bound by these factors. The KO enriched DARs on the other hand, showed enrichment of DNA motifs normally bound by genomic architectural protein CTCF as well as bZIP (FRA1, BATF, JUNB, AP-1, FOSL2 and BACH2), STATs and NF-**κ**B transcription factors, which is in line with enhanced inflammatory gene signatures observed in Arid1a-deficient B cells (Figure 4G).

We next examined the association between changes in gene expression and alterations in chromatin accessibility in Arid1a-deficient B cells. Our analysis identified a clear overlap (~31%) between downregulated DEGs and control DARs, which showed diminished accessibility upon Arid1a loss. Though in stark contrast, very few (~4%) upregulated DEGs harbored DARs that gained accessibility in Arid1a KO B cells (Figure 4H). Among specific examples, the *Il6, Ccl2, Ccl7* gene loci and the interferon-induced tetratricopeptide repeats (*Ifit)* gene cluster showed increased gene expression linked with nearby regions with enhanced chromatin accessibility expression (Figure 4I–J, S5E). On the other end of the spectrum, *Tigit* (and *Prdm1*, not statistically significant) locus showed reduced accessibility signals and decreased expression (Figure 4I–J, S5E). It is worth noting, that the transcripts encoding IgD and IgM isotypes (*Ighd, Ighm*) were upregulated in Arid1a-deficient B cells and this correlated with a slight increase in accessibility near the *Ighm* locus (Figure 4I (lower right panel)). This increase in mRNA levels of *Ighd, Ighm* explains the elevated cell surface protein expression of IgM and IgD isotypes observed in Arid1a-deficient B cells. Conversely, gene encoding IgE isotype (*Ighe*) was transcriptionally repressed and was associated with decreased accessibility near the *Ighe* locus overlapping regions of 3’ regulatory region (3’RR) of the IgH locus (Figure 4J (lower right panel)). In sum, these analyses reveal that Arid1a-dependent cBAF activity is crucial for establishing accessibility at regions associated with B cell activation and simultaneously plays an important role in suppressing inflammatory gene and accessibility signatures.

### Arid1a-deficient B cells initiate the GC response but fail to sustain GCs

**Figure 5:**
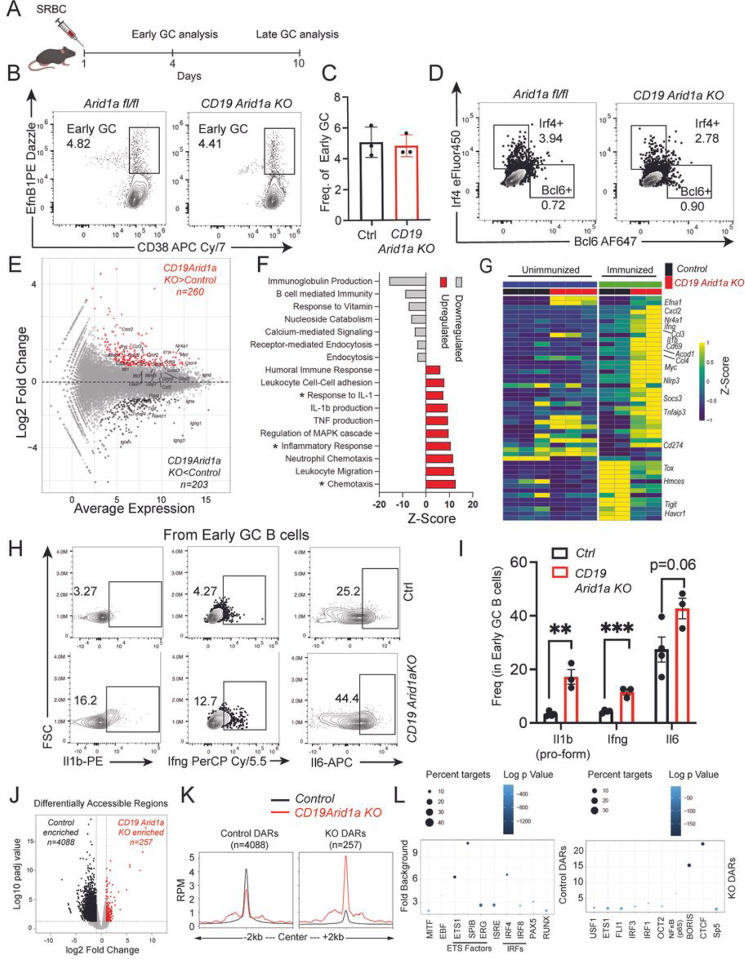
Arid1a-deficient B cells fail to sustain GCs. **A.** Schematic for immunization with SRBC for *Arid1a fl/fl* and *CD19 Arid1a KO* for early (day 4) and late GC (day 10) analysis. **B.** Representative flow cytometry analysis of early GC B cells (EfnB1+CD38+) gated on splenic B cells from SRBC immunized *Arid1a fl/fl* and *CD19 Arid1aKO* mice at day **4** post-immunization. **C.** Quantification of early GC B cell frequency in spleen of *Arid1a fl/fl* (YFP-) and *CD19 Arid1a KO* (YFP+) mice at day 4 post-immunization. **D.** Representative flow cytometry plots for Irf4 and Bcl6 gated on splenic B cells from day 4 SRBC immunized *Arid1a fl/fl* and *CD19 Arid1aKO* mice. **E.** MA plot of RNA-sequencing data displaying changes in gene expression in *CD19 Arid1aKO* B cells compared to control B cells. The highlighted genes (Red: upregulated, Black: downregulated) are differentially expressed genes. **F.** Upregulated (red) and downregulated (grey) pathways in *CD 19 Arid1aKO* compared to control B cells. The *x*-axis represents the Z-score, and asterisks highlight relevant pathways from Metascape analysis. **G.** Heatmap of gene expression data for *CD19 Arid1a KO* and *control* early GC B cells and B cells from a subset of differentially expressed genes with or without immunization. The color palette represents the Z-score from the TPM values. **H.** Representative flow cytometry analysis of Il1 b (pro-form), Ifng and Il6 in Control (Ctrl) (YFP-) and *CD19 Arid1aKO* (YFP+) early GC B cells (EfnB1 +CD38+) in spleen at day 4 following SRBC immunization. **I.** Quantification of Control (Ctrl) (YFP-) and *CD19 Arid1aKO* (YFP+) early GC B cell frequency for expression of Il1b (pro-form), Ifng and Il6 proteins at day 4 post-immunization. **J.** Volcano plot (top) of ATAC-seq data displaying differentially accessible regions (DARs) in *CD19 Arid1a KO* compared to control early GC B cells. **K.** Profile histograms showing the mean ATAC-seq signal in 6507 control and 570 KO enriched DARs (*control* B cell signal, black; *CD19 Arid1aKO* signal, red). **L.** Motif enrichment analysis of control DARs (top) and KO DARs (bottom) from *CD19 Arid1a KO* and *control* early GC B cells. The *y*-axis indicates the fold enrichment over background, circle size indicates the percentage of regions with the respective motif, and the color indicates the significance (log_10_ *P* value). Statistical significance is calculated using two-tailed Student’s t-test for C and L. Error bars represent mean ± s.e.; *p value ≤ 0.05; **p value ≤ 0.01; ***p value ≤ 0.0005; ****p value < 0.0001. Please see bioinformatics analysis in methods for additional information.

Since Arid1a-deficient B cells could be efficiently activated *in vitro*, we postulated that the failure to form GCs *in vivo* could be due to defects in sustenance of the GC B cell program. To this end, we immunized *CD19 Arid1a KO* and *Arid1a fl/fl* mice with SRBC and analyzed them at early stages (day 4) of GC induction (Figure 5A). Intriguingly, at day 4 post-immunization, the early-stage pre-GC B cells (early GC B cells; CD38+ Efnb1+ or PNA+) were comparable between Arid1a-deficient and control mice (Figure 5B–C, S6A-B). These early GC B cells were present even at day 10 post-immunization with SRBCs (Figure S6C). Importantly, at these early stages, Arid1a-deficient B cells expressed Irf4 and Bcl6, which are key transcription factors required for B cell activation and GC induction (Figure 5D, S6D-E). Besides, the T follicular helper (Tfh) cell subset, which requires input signals from activated B cells for their differentiation, were also comparably induced in *CD19 Arid1a KO* and *Arid1a fl/fl* mice at day 4 post-immunization (Figure S6F-G), and the Tfh pools were maintained even at late stages of immunization with SRBC and NP-Ova (in both *CD19 Arid1a KO* and *Cg1 Arid1a KO* mice) (Figure S6H-L). As noted earlier, by day 10 post-immunization with SRBCs the mature GC B cell frequencies were greatly reduced in *CD19 Arid1a KO* mice (Figure S6C). Interestingly, despite the strong absence of GCs at late stages of immunization in *CD19 Arid1a KO* mice, the frequency of antigen specific total (NP+ CD38+ IgDlo; Figure S7A-D) and class switched (NP+ CD38+ IgDlo IgG1+; Figure S7E) memory B cells were not significantly altered upon NP-Ova immunization, compared to controls. Altogether, these studies provide clear evidence that GC maintenance rather initiation is the major defect in Arid1a-deficient B cells.

To interrogate this in a more detailed manner, we performed RNA-seq and ATAC-seq in early GC B cells (CD38+ Efnb1+ GL7+ CCR6+) sorted from *CD19 Arid1a KO* and *Arid1a fl/fl* mice 4 days post-immunization with SRBCs (in biological replicates). RNA-Seq revealed 260 upregulated and 203 downregulated genes (p value ≤0.05; log2 fold change greater than +/− 1) in Arid1a-deficient pre-GC B cells in comparison to control cells (Figure 5E). Both *Bcl6* and *Irf4* were induced in the Arid1a-deficient B cells, and the accessibility profiles at these loci were comparable to control cells, which suggests an efficient induction of GC program (Figure S7F-G). Remarkably, in strong congruence to our findings in Arid1a-deficient B cells stimulated *in vitro*, the Arid1a-deficient early GC B cells showed upregulation of inflammatory gene signatures, including expression of cytokine (*Il1b, Ifng*), chemokine (*Ccl3, Ccl4, Cxcl2*), chemokine receptor (*C5ar1,Cxcr2,Cxcr4,Ccr5,Csf3r)*, cell activation (*Myc, Nr4a1, Cd69)* as well as interferon stimulated (*Ifit1, Ifit3, Ifitm3, Oasl2, Gbp2, Gbp5*) genes (Figure 5E–G, S7H-K). The downregulated genes were represented by negative regulators of B cell receptor signaling, such as *Tigit, Havcr1, Pdcd1* and *Tox* (Figure 5E,5G, S7J). Importantly, these changes in gene expression were mainly evident in activated Arid1a-deficient pre-GC B cells and were largely absent from Arid1a-deficient naive B cells (Figure 5G). Moreover, we confirmed striking increase in percentage of Arid1a-deficient early GC B cells expressing high levels of Il1b (~6-fold higher), Ifn-gamma (Ifng: ~3-fold higher) and Il6 (slightly higher) by flow cytometry compared to control cells (Figure 5H–I), and these changes were specific to activated B cells and missing in non-GC B cells (Figure S7L). GSEA further revealed a significant enrichment of acute inflammatory and chemotaxis pathways in Arid1a-deficient early GC B cells (Figure S7M). In addition to enhanced gene signatures mediating lymphocyte chemotaxis, the GSEA identified a surprising enrichment of gene sets associated with monocyte chemotaxis in Arid1a-deficient early GC B cells (Figure S7M).

The ATAC-seq analysis of Arid1a-deficient pre-GC B cells found a global decrease in chromatin accessibility, with the majority of regions (n=4088; controls DARs) displaying diminished accessibility signal and a small fraction (n=257; KO DARs) gaining accessibility (Figure 5J–K, S7N). Like our observations with *in vitro* stimulated Arid1a-deficient B cells, the control DARs from early GC B cells harbored motifs associated with EBF, ETS, IRF (IRF4 and IRF8), PAX5 and RUNX binding, whereas the KO DARs showed enrichment of IRF1, IRF3, NF-**κ**B, some ETS and CTCF motifs (Figure 5L). Almost 25% of all KO DARs contained a CTCF motif, thus unveiling strong enrichment of CTCF binding signatures in Arid1a-deficient activated B cells under both *in vitro* and *in vivo* stimulations. The control DARs were located further away from the promoters (median distance ~25Kb from TSS), and the KO regions again showed broader accessibility profiles and were associated with more promoter proximal regions (median distance ~11kb from TSS) (Figure 5K). Altogether, these results demonstrate that Arid1a-deficient B cells initiate GC responses *in vivo*, but fail to sustain them, and analogous to their *in vitro* stimulated counterparts, Arid1a deficiency in early GC B cells instigates inflammatory programs.

### Arid1a deficiency in B cells is associated with enhanced recruitment of inflammatory myeloid cells

**Figure 6:**
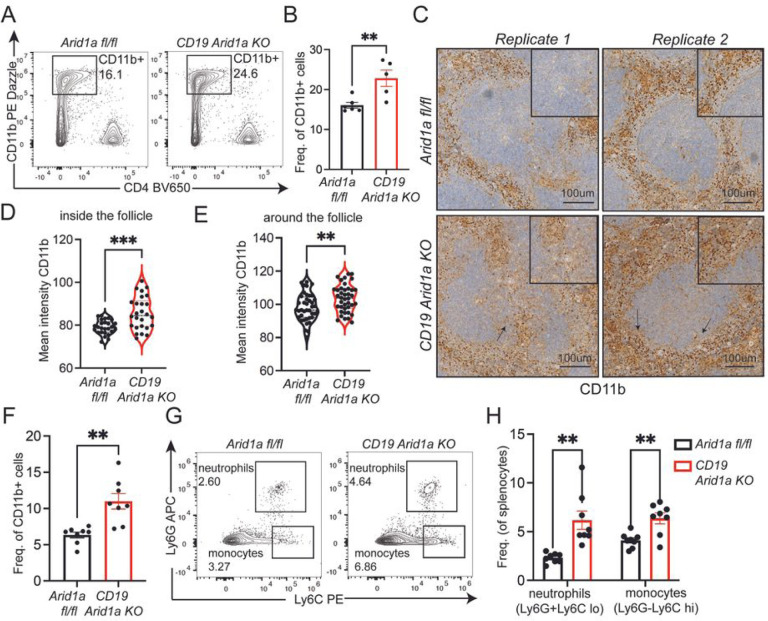
Arid1a deficiency in B cells promotes infiltration of inflammatory myeloid cells. **A.** Representative flow cytometry analysis of splenic CD11b+ myeloid cells from NP-Ova immunized *Arid1a fl/fl* and *CD19 Arid1aKO* mice at day 14 post-immunization. **B.**Quantification of CD11b+ myeloid cells frequency in spleen of *Arid1a fl/fl* and *CD19 Arid1a KO* mice at day 14 post-immunization. **C.**Representative immunohistochemistry images for CD11b staining on formalin fixed spleen sections from *Arid1a fl/fl* and *CD19 Arid1a KO* mice immunized with NP-Ova at day 14 post immunization. Magnified inlet from replicate 1 (both *Arid1a fl/fl* and *CD19 Arid1a KO,* left panels) shows CD11b staining inside the B cell follicles. The magnified inlet from replicate 2 (right panels) shows CD11b staining around the B cell follicles. **D-E.** Violin plot quantifying the mean intensities of CD11b staining using Image J software for both inside (**D**) and around (**E**) the follicle. **F.** Quantification of CD11b+ myeloid cell frequency by flow cytometry in spleen of *Arid1a fl/fl* and *CD19 Arid1a KO* mice at day 4 post-immunization. **G.** Representative flow cytometry plots showing neutrophils (Ly6G+Ly6C lo) and inflammatory monocytes (Ly6G-Ly6C hi) from day 4 SRBC immunized *Arid1a fl/fl* and *CD19 Arid1aKO*spleens. **H.** Quantification of neutrophils (Ly6G+Ly6C lo) and inflammatory monocytes (Ly6G-Ly6C hi) from *Arid1a fl/fl* and *CD19 Arid1aKO* mice at day 4 post-immunization. Statistical significance is calculated using two-tailed Student’s t-test for B, and D-F, multiple unpaired t-tests for H. Error bars represent mean ± s.e.; *p value ≤ 0.05; **p value ≤ 0.01; ***p value ≤ 0.0005; ****p value < 0.0001.

The enhanced inflammatory signatures, including the upregulation of Il1b, Ifng, Il6 and pathways associated with monocyte chemotaxis in Arid1a-deficient B cells, compelled us to formally evaluate possible changes in inflammatory cell populations within the myeloid compartment. A recent study has shown that bacterial infections disrupt ongoing GCs through recruitment of inflammatory monocytes^23^. Interestingly, the frequency of CD11b+ myeloid cells were significantly increased in NP-Ova immunized *CD19 Arid1a KO* in comparison to *Arid1a fl/fl* control mice (Figure 6A–B). NP-Ova immunized Cg1 Arid1a KO mice also showed a trend towards an increase in myeloid cells, though it did not reach statistical significance (Figure S8A-B). The immunohistochemistry analysis of *CD19 Arid1a KO* mice immunized with NP-Ova also revealed elevated presence of CD11b+ myeloid cells in the red pulp and intense infiltration of CD11b+ myeloid cells within the B cell follicles (Figure 6C–E, S8E). Unlike *Arid1a fl/fl* mice, many B cell follicles in the spleen of *CD19 Arid1a KO* mice displayed pronounced infiltration by CD11b+ cells (Figure 6C, 6E), and the histological architecture particularly around the edges of B cell follicles was severely deformed (Figure 6D, 6E). The increased infiltration by CD11b+ myeloid cells was evident even at early stages of immunization with SRBCs in *CD19 Arid1a KO* mice (Figure 6F). Within the myeloid cell populations, we observed an increase in frequency and numbers of Ly6G+ neutrophils and Ly6C high inflammatory monocytes in *CD19 Arid1a KO* mice compared to immunized control mice at both early (day 4) and late stages (day 10) (Figure 6G–H, S8C-E). Thus, indicating that the inflammatory signals upregulated upon Arid1a deficiency in activated B cells promotes increased infiltration by inflammatory myeloid cell subsets.

### Dampening of inflammatory signals rescue GC B cell differentiation of Arid1a-deficient cells.

While Arid1a deficiency disrupts GC homeostasis, we did not identify major defects in known transcriptional, epigenetic or signaling regulators of GC differentiation that could explain the dramatic collapse of GCs at later stages. Therefore, we asked if the loss of GCs could be a consequence of enhanced inflammatory signatures which were consistently observed in Arid1a-deficient B cells. Since the inflammatory signals emanating from Arid1a-deficient B cells are quite multi-faceted, we decided to use the glucocorticoid receptor (GR) agonist, Dexamethasone (Dexa) as a broad anti-inflammatory agent to blunt these signals^27^. Importantly, glucocorticoids and their agonists function by trans-repression mechanisms of co-factor sequestration to potently suppress NF-**κ**B, IRF and AP-1 signaling^27–29^. The NF-**κ**B, IRF and AP-1 signaling are molecular signatures enriched in Arid1a-deficient B cells, providing additional rationale for testing this approach.

**Figure 7:**
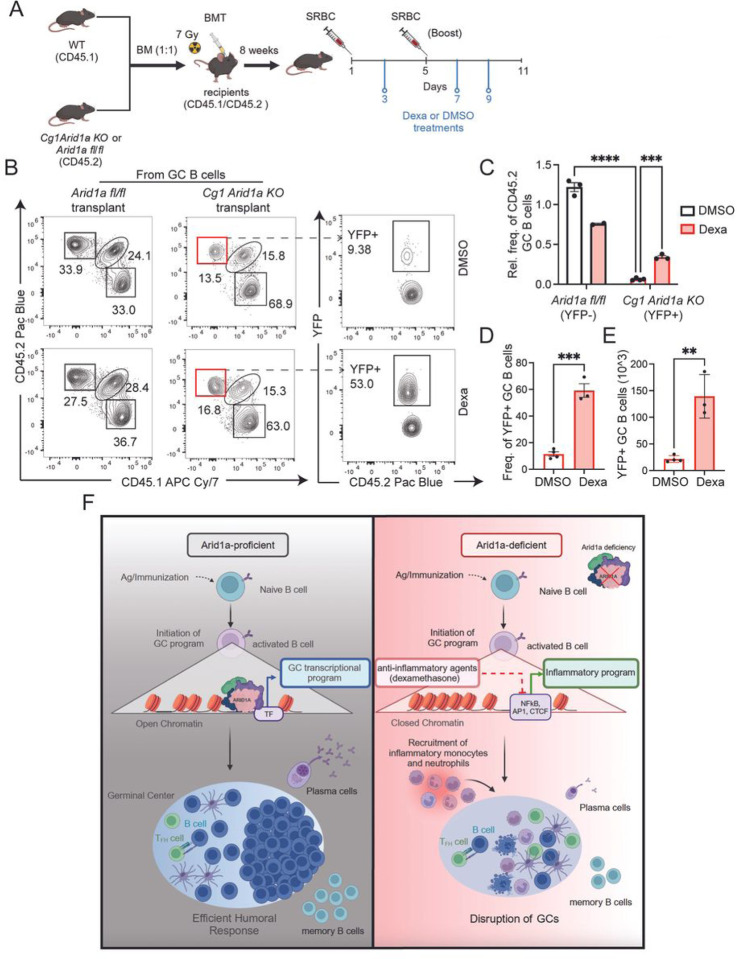
Dampening of inflammatory signals rescues GC differentiation in *Arid1a*-deficient B cells. **A.** Schematic for bone marrow transfer (BMT) experiment for evaluating the effects of Dexa on GC B cells from either CD45.2 expressing *Arid1a fl/fl or Cg1 Arid1aKO* bone marrow (BM) chimeras in CD45.1.2 expressing recipients. **B.**Representative flow cytometry analysis depicting the distribution of splenic CD45.2 GC B cells (redboxes) and CD45.1 GC B cells (dark grey boxes) within total GC B cells from SRBC immunized *Arid1a fl/fl* and *Cg1 Arid1aKO*chimeras at day 11 post-immunization with DMSO or Dexa treatments. The distribution of YFP+ GC B cells from *Cg1 Arid1a KO* (CD45.2) chimeras treated with DMSO (*top*) or Dexa (*bottom*) are shown on the right, see dashed arrows. **C.** Quantification of splenic CD45.2 GC B cell frequency normalized to CD45.1 GC B cells within the same mice from *Arid1a fl/fl* (YFP-) and *Cg1 Arid1aKO* (YFP+) chimeras at day 11 post-immunization and respective (DMSO and Dexa) treatments. The frequency of each subset was calculated from the live cell gate to account for differences in engraftment. *Arid1a fl/fl* and *Cg1 Arid1aKO* chimeras treated with DMSO are open bars and Dexa treated groups are represented with light red filled bars. **D and E.** Quantification of YFP frequency **(C)** and absolute numbers **(D)** gated on CD45.2 GC B cells from *Arid1a fl/fl or Cg1 Arid1aKO* chimeras. **F.** Proposed model by which *Arid1a* regulates GC responses. *On left,* Arid1a mediates the establishment of chromatin landscapes required for GC transcriptional program. *On right,* Arid1a deficiency instigates an inflammatory transcriptional program which recruits inflammatory cell types leading to premature GC collapse. Statistical significance is calculated using 2-way ANOVA with multiple comparisons for C and two-tailed Student’s t-test for D and E. Error bars represent mean ± s.e.; *p value ≤ 0.05; **p value ≤ 0.01; ***p value ≤ 0.0005; ****p value < 0.0001.

For these studies, we generated bone marrow chimeras with congenic CD45.1 expressing wildtype cells mixed with CD45.2 expressing *Arid1a fl/fl* or *Cg1 Arid1a KO* cells transplanted into sub-lethally irradiated CD45.1.2 recipients. 8 weeks following bone marrow transfer, we confirmed the establishment of stable chimeras and immunized mice with SRBCs to induce GC responses. These mice were additionally treated with Dexa or DMSO (as control), at days 3, 7 and 9 after initial SRBC immunization and spleens were harvested at day 11 for analysis (Figure 7A). To account for possible differences in engraftment efficiencies and immunization variabilities between mice, we quantified ratios of CD45.2 B cells relative to CD45.1 B cells within the same mice. To begin, the relative frequency of CD45.2 B cell was not significantly altered in DMSO or Dexa treated *Cg1 Arid1a KO* chimeras, compared to *Arid1a fl/fl* chimeras, demonstrating no substantial changes in total B cell distribution in these transplanted groups (Figure S9A-B). As expected, within the GC B cell pool we observed a strong depletion of *Cg1 Arid1a KO* CD45.2 GC B cells (YFP+) in comparison with *Arid1a fl/fl* CD45.2 GC B cells in DMSO treated groups (Figure 7B–C), demonstrating loss of Arid1a-deficient GC B cells even in this context. In addition, the relative frequency of bystander CD45.1.2 GC B cells (normalized to CD45.1 GC B cells) was also significantly reduced in *Cg1 Arid1a KO* chimeras compared to *Arid1a fl/fl* control chimeras in the DMSO treated groups, thus revealing detrimental effects of Arid1a-deficient (activated) GC B cells on their wildtype counterparts (Figure S9C). These results imply that the cell-extrinsic effects of inflammatory signals from Arid1a-deficient activated B cells suppress GC responses in wildtype cells.

Notably, Dexamethasone (Dexa) treatment led to a significant increase in the overall contribution of *Cg1 Arid1a KO* (YFP+) cells to the GC B cell pool upon immunization, compared to the *Cg1 Arid1a KO* chimeras treated with DMSO (Figure 7B–E). Dexa treatment in immunized *Cg1 Arid1a KO* chimeric mice induced an ~5.5 fold increase in both frequency and absolute numbers of *Cg1 Arid1a KO* YFP+ GC B cells (Figure 7B, 7D–E). This effect is particularly remarkable considering that the treatment with Dexa causes an overall decrease in GC B cell frequency (compared to DMSO) in wildtype mice immunized with SRBCs (Figure S9D-E).

In comparison with *Arid1a fl/fl* control chimeras, the *Cg1 Arid1a KO* chimeras also displayed an increase in the frequency and numbers of Tfh cells and Cd11b expressing myeloid cells, including inflammatory monocytes and neutrophils (all within CD45.1 expressing lineages) (Figure S9 F-N). While treatment with Dexa led to a slight but significant decrease in the total splenocyte numbers (Figure S9F), it did not significantly alter the distribution of these myeloid cell populations in *Cg1 Arid1a KO* transplanted mice for the most part (Figure S9F,G-N). The only changes we observed upon dexa treatment were decrease in the frequency and numbers of Tfh cells (Figure S9G-H), and a slight increase in the frequency of neutrophils (Figure S9M). From these findings it seems that the rescue of Arid1a-deficient GC B cells upon dexa treatment primarily occurs through a B cell intrinsic mechanism. However, we cannot rule out qualitative functional changes in infiltrating myeloid and Tfh populations following treatment with dexa. From these studies, we conclude that dampening of inflammatory signals by the GR agonist, Dexamethasone, partially rescues the GC differentiation of Arid1a-deficient GC B cells. Moreover, these findings underscore the importance of Arid1a-mediated suppression of inflammatory signals in promoting efficient humoral responses.

## Discussion

Germinal centers are essential for generation of “high-quality” humoral immune responses^16–18^, and our findings presented here identify a critical role for the Arid1a-dependent cBAF activity in orchestrating epigenetic and transcriptional transitions needed for generation of effective GC B cell responses. We show that even though Arid1a-deficient B cells undergo initial activation (both *in vitro* and *in vivo*), they adopt “type1-like” inflammatory signatures, which contributes to perturbations in transcriptional fidelity leading to GC disruption (Figure 7F). Our results accentuate the importance of elevated inflammatory signatures caused by Arid1a deficiency in GC collapse, as blunting of these signals by anti-inflammatory GR signaling, at least partially rescued GC B cell differentiation in Arid1a-deficient B cells (Figure 7F). We further show that the unconstrained inflammation not only perturbs GC homeostasis in Arid1a-deficient B cells, but also disrupts GC responses in wildtype cells, highlighting both cell-intrinsic and -extrinsic roles of Arid1a-loss associated inflammatory signals in regulating GC responses. Importantly, Arid1a deficiency in activated B cells causes broad epigenetic and transcriptional alterations, and we could not simply ascribe the defects in GC B cell differentiation to deregulation of known regulators of the GC program. Therefore, we believe that the GC phenotypes associated with Arid1a in B cells are likely a consequence of a multitude of molecular and cellular changes, such as the inflammatory signatures that we describe here. Taken together, our studies reveal critical functions of the Arid1a-dependent cBAF activity in suppressing inflammation and preserving efficient GC responses.

Other recent studies have also described negative effects of unrestrained inflammation on GC responses. For instance, Salmonella and Listeria infections during ongoing GC responses have been shown to disrupt GCs through an IFN*γ*-dependent recruitment of monocytes^23^. Similarly, in COVID patients with systemic inflammation and severe infection the conventional GC responses appear to be interrupted, even though patients with milder COVID infection generate robust GC responses^24,25^. In our own studies, we observed strong upregulation of IFN*γ*, Il1b (as well as Il6 and several chemokines), which are key regulators involved in recruitment and activation of inflammatory innate immune cells. These observations and our studies here pose the question, whether strong inflammatory responses can actively dictate the quality of adaptive immune response. With the uncoupling of inflammatory and adaptive immune responses, the implementation of such feedback control could offer the potential advantage of preventing the initiation of GC response in highly inflamed and damaged tissues. This in turn, could safeguard against autoimmunity. It will be fascinating to find similar themes emerging in other conditions associated with heightened inflammatory states such as pathogen infections and inflammatory diseases.

Ligand-bound glucocorticoid receptors (GRs) translocate to the nucleus where they function through multiple mechanisms to blunt inflammation^27–29^. One of the well-described phenomena by which GRs act is trans-repression, where ligand-bound active GRs sequester transcriptional co-activators away from NF-**κ**B, IRF and AP-1 transcription factors to repress inflammation-linked gene programs^27–31^. Interestingly, DNA binding motifs for these transcription factors were strongly enriched among KO-specific DARs that gained accessibility upon Arid1a loss. In our experiments, dexamethasone treatment rescued Arid1a-deficient GC B cell differentiation without altering the distribution of myeloid cell populations. Though we cannot rule out functional changes in myeloid cell populations upon dexamethasone treatment, based on these observations, it is likely that dexamethasone primarily functions through inhibiting pro-inflammatory signals within Arid1a-deficient B cells. Notably, previous studies have also described a physiological role for glucocorticoids in regulating antibody responses^32^. While further investigation of GR biology in regulating GC B cell responses is beyond the scope of our present studies, we intend to follow up on precise mechanisms by which GR suppresses inflammation in the context of Arid1a deficiency in future work.

Our data shows that the ETS, EBF and IRF family of transcription factors which are required for normal B cell activation and differentiation, rely on the Arid1a-dependent cBAF complex for remodeling chromatin landscapes. In addition, we uncover important functions of the Arid1a associated cBAF complex in suppressing inflammatory gene programs through regulation of NF-**κ**B, IRF, AP-1 and CTCF activity, which are known to drive inflammatory gene networks^28,33^. Among the inflammatory changes, we noted elevated expression of several cytokines and chemokines following Arid1a depletion in B cells, which indicates the importance of Arid1a-dependent BAF complex in maintaining these loci in repressed states. Consistently, the cBAF complex is known to interact with histone deacetylases to remove acetylation and shut down transcription at various cytokine genes in ovarian cancer cells^6,34^. This could possibly explain the surprising lack of correlation between upregulated genes and changes in chromatin accessibility upon Arid1a deletion and may indicate a de-repression mechanism mediated by alterations in histone marks. That being said, additional mechanisms, such as compensation by other BAF complexes and regulation of 3D genomic architecture (through altered CTCF binding), may operate simultaneously to drive these inflammatory gene programs in Arid1a-deficient B cells^6,33,35^. Moreover, two previous studies in B cells had genetically disrupted Baf155 (Smarcc1) and the major ATPase subunit, Brg1 which are part of all three mammalian BAF (cBAF, PBAF and ncBAF) complexes^36,37^. Loss of Baf155 or Brg1 in these studies led to disruption of GCs as a result of specific changes in Prdm1 expression or cell cycle associated pathways, respectively, but these changes were not observed in our studies. This further emphasizes the functional heterogeneity in the activity of distinct BAF complexes and how they may regulate specific accessibility and gene expression programs even within the same cell type.

It is noteworthy that several recent studies have investigated the functions of different BAF complexes during T cell differentiation in the context of chronic viral infection and anti-tumor immunity^38–43^. Our work presented here has interesting parallels to several findings from these studies. Arid1a loss in CD8 T cells, does not affect generation of early effector cells, disrupts terminal effector cell differentiation and preserves circulating memory cells^40^. Highly consistent with phenotypes in T cells, our studies found early activation of B cells, decrease in terminally differentiated plasma cells, loss of GC B cells and no significant changes in antigen-specific memory B cells upon Arid1a loss. Furthermore, even on a genomic level Arid1a deficiency in CD8 T cells is associated with reduced accessibility at ETS and IRF motifs, and a gain in accessibility at AP-1 and CTCF sites, which is again in strong congruence with features of Arid1a deficiency in B cells^40^.

Lastly, while Arid1a mutations (and mutations in other cBAF subunits) are frequently associated with germinal center-derived lymphomas, Arid1a loss by itself was not sufficient to induce malignant transformation of B cells, even in mice that were aged up to 12 months (not shown). Most likely, Arid1a mutations cooperate with other genetic events to drive lymphomagenesis. In this context, it will be interesting to determine how the inflammatory signatures described here may contribute to oncogenesis associated with Arid1a mutations, including those seen in pre-malignant states such as clonal hematopoiesis. Though the initial trigger for inflammation in Arid1a-deficient B cells remains unclear, a recent study^44^ has reported similar enrichment of inflammatory pathways in gastric cancer patients with genetic alteration in Arid1a.

## Methods

### Mice

*Arid1a floxed* mice were generated as previously described^45^ and were obtained from the Jackson Laboratory (027717). These mice were crossed with *CD19 cre* or *Cg1 cre* to generate *CD19 Arid1a KO* or *Cg1 Arid1a KO*. The *Cre* expressing mice were crossed with *Rosa26-LSL-YFP* reporter mice, described previously^46^. The CD45.1 mice (002014, *Ptprc*^*a*^ Pep Boy) and *CD19 cre* (006785) were obtained from Jackson Laboratory. All mice were maintained on a C57/Bl6 genetic background. Both male and female mice aged between 6 to 12 weeks were used in the experiments. Animals were bred and housed at the specific pathogen free facility at Northwestern University and all protocols and experimental procedures were approved by Institutional Animal Care and User Committee guidelines.

### Immunizations

For NP-Ova and SRBC immunizations, animals were immunized intraperitoneally (*i.p*) For NP-Ova, 50 μg NP-Ova (#N-5051–10, Biosearch Technologies) was mixed with the imject alum as an adjuvant (#77161, Thermo Scientific) at a 1:1 ratio and was mixed overnight at 4°C on a rotator. The adjuvanted NP-Ova was injected at day 1 (prime) and day 5 (boost), to induce more consistent GC responses. For SRBC immunizations, citrated SRBCs (Colorado Serum Company) were washed twice with PBS. Mice were first primed with a low dose (200 × 10^6^ SRBCs) on day 0 followed by a boost on day 5 with a higher dose (10^9^ SRBCs). Mice were analyzed on day 14 post the first immunization. For the early GC analysis, mice were immunized only once with a high dose (10^9^ SRBCs), and spleens were collected at Day 4 post immunization.

### Antibody Measurement Assays

To measure antibody titers, mice were bled on Day 0 and Day 14, post-immunization. Antibody titers were determined by ELISA. High binding assay plates were coated with either 50 μL of NP36-BSA (10 μg/mL, Biosearch) or NP2-BSA (10 μg/mL, Biosearch) overnight at 4 °C. Plates were blocked for 2 h at 37 °C with 50 μL of 5% BSA and 0.05% sodium azide in PBS. Blocked plates were then washed twice with deionized water After washing, 50μL mouse serum samples (diluted in 1% BSA and 0.1% Tween-20 in PBS) were added and incubated for 2 h at 37°C. Plates were washed and incubated with alkaline phosphatase–conjugated detection IgM and IgG1 antibodies (Southern Biotech) for 2 h. Plates were washed and incubated with 100 μL phosphatase substrate solution (Invitrogen) for 10–15 min, and the absorbance was measured at 405 nm using Spectramax M2 plate reader.

For SRBC-specific IgM and IgG1 antibody titers, a flow-cytometry based assay was used as previously described^47^. Briefly, SRBCs were incubated for 20 min on ice with 3-fold serial dilutions of the serum samples from SRBC immunized mice. The labelled SRBCs were then washed with the flow cytometry buffer (0.5% bovine serum albumin, 1 mM EDTA and 0.05% sodium azide in PBS), and stained with anti-mouse IgM and anti-mouse IgG1. Mean fluorescence intensities of the SRBCs bound anti-IgM and anti-IgG1 antibodies were plotted at different serum dilutions, and values in the linear range were used for the final presentation of the results.

### B cell isolation and 40LB co-cultures

For *ex vivo* expansion and differentiation of B cells, 40LB feeder cells were irradiated with 3000 cGy (x-rays or γ-rays). The irradiated cells were plated at a density of 50 × 10^4^ cells per well on a 6-well plate and were cultured overnight at 37 °C with 5% CO_2_ in DMEM supplemented with 10% FBS, 10 mM HEPES (pH 7.4), 2 mM GlutaMAX, 1 mM sodium pyruvate, and 55 μM 2-mercaptoethanol (all from Life technologies). The 40LB cells were procured from the D. Kitamura’s Laboratory (Tokyo University of Sciences). The 40LB cells were validated through B cell stimulation experiments and were regularly screened for mycoplasma contamination. Primary B cells were isolated using mouse B cell isolation kit (Cat#19854, Stem cell Technologies) from spleens of *Cγ1 cre* and *Cγ1 Arid1aKO* or *Arid1a fl/fl* and *CD19 Arid1aKO* mice. The isolated B cells were seeded at a density of 10 ×10^4^ cells per well on 40LB in a 6-well plate in RPMI 1640 medium prepared as above and supplemented with 1 ng/ml of rmIL-4. The expanded B cells collected from the suspension were analyzed by flow cytometry on Day 4. For plasma cell differentiation, 10 ×10^4^ B cells from Day 4 were replated on freshly irradiated 40LB feeder cells in the presence of IL-21 (10ng/mL). Cells were analyzed after 3 days of IL-21 treatment.

### Flow cytometry

Primary cells and *ex vivo*-cultured cells were stained in FACS buffer (0.5% bovine serum albumin, 1 mM EDTA and 0.05% sodium azide in PBS) with the appropriate antibodies for 30 min on ice. Cells were washed and then fixed with 4% paraformaldehyde (PFA; diluted to 1% with PBS; Affymetrix) for 10 min at RT before FACS analysis using Cytek Aurora (Cytek Bio). Antibodies were obtained from BioLegend, eBioscience and BD Pharmingen. For intracellular staining, cells were first fixed with 4% PFA for 10 mins at room temperature (diluted to 1% with PBS) and then permeabilized with Foxp3/Transcription factor staining buffer kit (eBioscience #00-5523-00) for at least 1 hour before staining with appropriate antibodies. Flow cytometry data was analyzed using the FlowJo (version 10.9.0) software. The gating strategy for the flow cytometric analysis in each experiment is described in the corresponding figures and legends.

### Immunoblotting

Proteins were isolated from B cells using NP-40 lysis buffer and were resolved using NuPAGE 4–12% Bis-Tris gels (Thermo Fisher) and transferred to PVDF membranes using iBlot^™^ 2 Gel Transfer system. Membranes were blocked with 5% non-fat dry milk in TBSTE buffer (50 mM Tris-HCl pH 7.4, 150 mM NaCl, 0.05% Tween 20, 1 mM EDTA) and incubated with primary antibodies followed by appropriate secondary antibodies conjugated with horseradish peroxidase (HRP). Arid1a/BAF250A (1:1000, clone D2A8U) and β-actin HRP (1:5000, clone 13E5) were purchased from Cell Signaling.

### RNA-seq library preparation, sequencing, and analysis

RNA from cells, obtained from 40LB co-cultures or flow cytometry sorted early GC B cells on Day 4 after SRBC immunization, were isolated using RNeasy Plus Mini Kit (Qiagen) following the manufacturer’s instructions. Purified RNA (100 ng) was subsequently utilized to prepare RNA-sequencing libraries with the NEBNext Ultra II Directional RNA Library Prep Kit (#E7760, New England Biolabs), following the manufacturer’s recommended protocol. Samples were sequenced on an Illumina Novaseq 6000 platform. Paired-end (50 bp) reads were mapped to the mouse genome mm10/GRCm38 using STAR^48^ (2.7.10a) (--genomeLoad LoadAndRemove --outFilterMismatchNmax 4 --outFilterMultimapNmax 100 --winAnchorMultimapNmax 100). Counts were obtained with featureCounts (subread v2.0.1)^49^ (-g gene_name -s 2 -S rf). Differentially expressed genes were calculated with DESeq2^50^, filtering out genes that did not have any count in any condition; cut off to define DEGs was an adjusted p-value 0.05 and a log_2_ fold change ≥ +/−1. The pathway enrichment analysis of DEGs was performed using Metascape (https://metascape.org).

### ATAC-seq library preparation, sequencing, and analysis

ATAC-seq was performed as described previously^51^. Briefly, 50,000 viable cells were obtained from either 40LB-stimulated or sorted early GC B cells on Day 4 after SRBC immunization. Cells were washed with cold PBS, collected by centrifugation, then lysed in resuspension buffer (RSB; 10 mM Tris-HCl, pH 7.4, 10 mM NaCl, 3 mM MgCl_2_) supplemented with 0.1% NP40, 0.1% Tween-20, and 0.01% digitonin. The samples were incubated on ice for 3 min, followed by a wash, \ with 1 mL cold RSB containing 0.1% Tween-20. Nuclei were pelleted by centrifugation at 500g for 10 min at 4 °C, resuspended in 50 μL transposition mix (25 μL 2x TD buffer, 2.5 μL transposase (100 nM at final concentration), 16.5 μL PBS, 0.5 μL 1% digitonin, 0.5 μL10% Tween-20 and 5 μL H_2_O), and incubated at 37 °C for 30 min. DNA was purified using Zymo DNA Clean and Concentrator-5 cleanup kit (#D4013, Zymo Research) and, then was PCR amplified (11–12 cycles) using indexed oligos. The resulting ATAC-seq libraries were purified once again using the Zymo DNA Clean and Concentrator-5 cleanup kit and sequenced using an Illumina NovoSeq 6000 platform.

Paired-end reads (50 bp) were mapped to the mouse genome mm10 GRCm38 (Dec. 2011) from UCSC using Bowtie 1.0.0^52^ (“--p 12 -m 1 --best --strata -X 2000 -S --fr --chunkmbs 1024”). Duplicated reads were marked and removed with picard-tools-2.21.4 MarkDuplicates (https://broadinstitute.github.io/picard/). Subsequently, these fragments were used to call peaks with HOMER (v4.9.1)^53^ using the findPeaks function for each replicate (-center -style dnase “). The intersected peaks from the same condition were obtained using HOMER mergePeaks and filtered with grep. The union of peaks from control and KO conditions were obtained with mergePeaks and was further used to identify differentially accessible regions (DARs) using MEDIPS^54^; DARs were defined by an adjusted p-value (FDR) lower than 0.05 and a log_2_ fold enrichment greater than or equal to 1.

### Bone Marrow transplantation

For transplantation experiments, whole BM cells from *Arid1a fl/fl* or *Cg1 Arid1a KO* (both CD45.2) were mixed in equal numbers with whole BM cells from CD45.1 mice (002014, *Ptprc*^*a*^; Jackson Laboratory). A total of 2×10^6^ BM cells/mouse (CD45.1 and CD45.2 BM mix) from *Arid1a fl/fl* control arm and *Cg1 Arid1a KO* mice were then transplanted into sub-lethally irradiated (700 cGy) CD45.1.2 congenic recipients through retro-orbital injections. The recipient mice were examined for engraftment at 8 weeks before immunizing them with SRBC for evaluating GC responses. Donor mice and recipients for transplantation studies were all females and were between 6–10 weeks of age at the time of BM transfer.

### Quantification and statistical analysis

Statistical analyses were performed with Graph Prism version 10.1.0. The statistical tests used to determine significance in each analysis are described in the respective figure legends of corresponding figures. Two-tailed student t-tests, multiple unpaired t-tests or 1- or 2-way ANOVA were used as appropriate. Results are displayed as mean ± standard error (se). No statistical methods were used to predetermine sample sizes. No data points were excluded from the analysis, and appropriate animals/samples for each experiment were chosen randomly. Since our analysis required genotyping of the experimental mouse groups, data acquisition was not blinded.

## Data Availability

All genome-wide sequencing datasets have been deposited to Gene Expression Omnibus (GEO) repository, accession number GSE246913. Any data and reagents will be made available upon request.
